# Supplementary material for: An analytic approximation of the feasible space of metabolic networks
Source: Nat Commun. 2017 Apr 6;8:14915. doi: 10.1038/ncomms14915 (PMC5384209; doi:10.1038/ncomms14915)
Supplement: Supplementary Information — Supplementary figures, supplementary notes and supplementary references. [file ncomms14915-s1.pdf]

Supplementary note 1. KL-divergence minimization of the full conditional probabilities

Let us now rewrite the full probability distributions in Eq. (15) and Eq. (10) making explicit the dependency of the normalization factors with respect to the parameters a_n, d_n:

$$Q^{(n)}\left(\boldsymbol{\nu}|\boldsymbol{b}\right) = \frac{1}{\tilde{Z}_{Q^{(n)}}} e^{-\frac{1}{2}(\boldsymbol{\nu}-\bar{\boldsymbol{\nu}})^T \boldsymbol{\Sigma}^{-1}(\boldsymbol{\nu}-\bar{\boldsymbol{\nu}})} \psi_n(\nu_n) \tag{S1}$$

$$Q\left(\boldsymbol{\nu}|\boldsymbol{b}\right) = \frac{1}{\tilde{Z}_Q\left(a_n, d_n\right)} e^{-\frac{1}{2}(\boldsymbol{\nu}-\bar{\boldsymbol{\nu}})^T \boldsymbol{\Sigma}^{-1}(\boldsymbol{\nu}-\bar{\boldsymbol{\nu}})} e^{-\frac{(\nu_n - a_n)^2}{2d_n}} \tag{S2}$$

where the partition functions are given by:

$$\tilde{Z}_{Q^{(n)}} = \int d^N \boldsymbol{\nu} e^{-\frac{1}{2}(\boldsymbol{\nu}-\bar{\boldsymbol{\nu}})^T \boldsymbol{\Sigma}^{-1}(\boldsymbol{\nu}-\bar{\boldsymbol{\nu}})} \psi_n(\nu_n) \tag{S3}$$

$$\tilde{Z}_Q\left(a_n, d_n\right) = \int d^N \boldsymbol{\nu} e^{-\frac{1}{2}(\boldsymbol{\nu}-\bar{\boldsymbol{\nu}})^T \boldsymbol{\Sigma}^{-1}(\boldsymbol{\nu}-\bar{\boldsymbol{\nu}})} e^{-\frac{(\nu_n - a_n)^2}{2d_n}} \tag{S4}$$

Let us compute $D_{KL}(Q^{(n)}||Q)$:

$$D_{KL}(Q^{(n)}||Q) = \int Q^{(n)}(\boldsymbol{\nu}|\boldsymbol{b}) \log\left(\frac{\psi_n\left(\nu_n\right)\tilde{Z}_Q\left(a_n, d_n\right)}{\phi_n\left(\nu_n\right)\tilde{Z}_{Q^{(n)}}}\right) d^N\boldsymbol{\nu} \tag{S5}$$

$$= \int Q^{(n)}(\boldsymbol{\nu}|\boldsymbol{b}) \log\left(\frac{\tilde{Z}_Q\left(a_n, d_n\right)}{e^{-\frac{(\nu_n - a_n)^2}{2d_n}}}\right) d^N\boldsymbol{\nu} + const \tag{S6}$$

$$= \int Q^{(n)}(\boldsymbol{\nu}|\boldsymbol{b}) \left[\frac{(\nu_n - a_n)^2}{2d_n} + \log\tilde{Z}_Q\left(a_n, d_n\right)\right] d^N\boldsymbol{\nu} + const \tag{S7}$$

$$= \frac{\langle(\nu_n - a_n)^2\rangle_{Q^{(n)}}}{2d_n} + \log\tilde{Z}_Q\left(a_n, d_n\right) + const \tag{S8}$$

where *const* does not depend on a_n and d_n. We aim at minimizing $D_{KL}(Q^{(n)}||Q)$ with respect to a_n, d_n:

$$\frac{\partial D_{KL}(Q^{(n)}||Q)}{\partial a_n} = \frac{-\langle\nu_n\rangle_{Q^{(n)}} + a_n}{d_n} + \frac{1}{\tilde{Z}_Q}\frac{\partial\tilde{Z}_Q}{\partial a_n} \tag{S9}$$

$$\frac{\partial D_{KL}(Q^{(n)}||Q)}{\partial d_n} = -\frac{\langle(\nu_n - a_n)^2\rangle_{Q^{(n)}}}{2d_n^2} + \frac{1}{\tilde{Z}_Q}\frac{\partial\tilde{Z}_Q}{\partial d_n} \tag{S10}$$

Since we can move the derivative inside the integration in $\frac{\partial\tilde{Z}_Q}{\partial a_n}$ and in $\frac{\partial\tilde{Z}_Q}{\partial d_n}$ we get:

$$\begin{aligned}\frac{1}{\tilde{Z}_Q}\frac{\partial\tilde{Z}_Q}{\partial a_n} &= \frac{1}{\tilde{Z}_Q}\int d^N\boldsymbol{\nu} e^{-\frac{1}{2}(\boldsymbol{\nu}-\bar{\boldsymbol{\nu}})^T \boldsymbol{\Sigma}^{-1}(\boldsymbol{\nu}-\bar{\boldsymbol{\nu}})} e^{-\frac{(\nu_n - a_n)^2}{2d_n}} \frac{(\nu_n - a_n)}{d_n} \\ &= \langle\frac{\nu_n - a_n}{d_n}\rangle_Q\end{aligned}$$

$$\begin{aligned}\frac{1}{\tilde{Z}_Q}\frac{\partial\tilde{Z}_Q}{\partial d_n} &= \frac{1}{Z_Q}\int d^N\boldsymbol{\nu} e^{-\frac{1}{2}(\boldsymbol{\nu}-\bar{\boldsymbol{\nu}})^T \boldsymbol{\Sigma}^{-1}(\boldsymbol{\nu}-\bar{\boldsymbol{\nu}})} e^{-\frac{(\nu_n - a_n)^2}{2d_n}} \frac{(\nu_n - a_n)^2}{2d_n^2} \\ &= \frac{\langle(\nu_n - a_n)^2\rangle_Q}{2d_n^2}\end{aligned}$$

Setting the derivatives in (S10) to 0 and assuming $d_n \neq 0$ we finally get

$$\begin{cases} 0 & = \frac{-\langle \nu_n \rangle_{Q^{(n)}} + a_n}{d_n} + \frac{\langle \nu_n \rangle_Q - a_n}{d_n} \\ 0 & = -\frac{\langle (\nu_n - a_n)^2 \rangle_{Q^{(n)}}}{2d_n^2} + \frac{\langle (\nu_n - a_n)^2 \rangle_Q}{2d_n^2} \end{cases} \tag{S11}$$

$$\begin{cases} \langle \nu_n \rangle_{Q^{(n)}} & = \langle \nu_n \rangle_Q \\ \langle \nu_n^2 \rangle_{Q^{(n)}} & = \langle \nu_n^2 \rangle_Q \end{cases} \tag{S12}$$

and thus the moment matching condition in Eq. (13) turns out to be equivalent to the KL-divergence minimization condition.

Supplementary note 2. Moments of the tilted distribution

Let us compute $\langle \nu_n \rangle_{Q^{(n)}}$ and $\langle \nu_n^2 \rangle_{Q^{(n)}}$ as

$$\langle \nu_n \rangle_{Q^{(n)}} = \frac{1}{\tilde{Z}_{Q^{(n)}}} \int d\nu_n \nu_n \psi_n(\nu_n) \int \prod_{m \neq n} d\nu_m e^{-\frac{1}{2}(\boldsymbol{\nu} - \bar{\boldsymbol{\nu}})^T \boldsymbol{\Sigma}^{-1} (\boldsymbol{\nu} - \bar{\boldsymbol{\nu}})} \tag{S13}$$

$$\langle \nu_n^2 \rangle_{Q^{(n)}} = \frac{1}{\tilde{Z}_{Q^{(n)}}} \int d\nu_n \nu_n^2 \psi_n(\nu_n) \int \prod_{m \neq n} d\nu_m e^{-\frac{1}{2}(\boldsymbol{\nu} - \bar{\boldsymbol{\nu}})^T \boldsymbol{\Sigma}^{-1} (\boldsymbol{\nu} - \bar{\boldsymbol{\nu}})} \tag{S14}$$

Integrating out the multivariate Gaussian we obtain for the first moment

$$\langle \nu_n \rangle_{Q^{(n)}} = \frac{1}{\tilde{Z}_{Q^{(n)}}} \int d\nu_n \nu_n q_n(\nu_n) \tag{S15}$$

where $q_n(\nu_n)$ is the marginal probability function

$$q_n(\nu_n) \propto \psi_n(\nu_n) e^{-\frac{(\nu_n - \bar{\nu}_n)^2}{2\Sigma_{nn}}} \tag{S16}$$

$$\propto \begin{cases} e^{-\frac{(\nu_n - \bar{\nu}_n)^2}{2\Sigma_{nn}}} & \text{if } \nu_n \in \left[v_n^{inf}, \nu_n^{sup}\right] \\ 0 & \text{otherwise} \end{cases} \tag{S17}$$

Let us rewrite the non-zero part of (S16) in standard notation:

$$q_n(\nu_n) = \frac{\frac{1}{\Sigma_{nn}} \mathcal{N}\left(\frac{\nu - \bar{\nu}_n}{\sqrt{\Sigma_{nn}}}\right)}{\Phi\left(\frac{\nu_n^{sup} - \bar{\nu}_n}{\sqrt{\Sigma_{nn}}}\right) - \Phi\left(\frac{\nu_n^{inf} - \bar{\nu}_n}{\sqrt{\Sigma_{nn}}}\right)} \tag{S18}$$

where the $\mathcal{N}(x) = \frac{1}{\sqrt{2\pi}} e^{-\frac{x^2}{2}}$ is the probability density function of the standard normal distribution and $\Phi(x) = \int_{-\infty}^{x} \frac{e^{-\frac{y^2}{2}}}{\sqrt{2\pi}} dy = \frac{1}{2}\left[1 + \text{erf}\left(\frac{x}{\sqrt{2}}\right)\right]$ is its cumulative. In the following we will need the value of the first two moments of this distribution that are given by:

$$\langle \nu_n \rangle_{Q^{(n)}} = \bar{\nu}_n + \frac{\mathcal{N}\left(\frac{\nu_n^{inf} - \bar{\nu}_n}{\sqrt{\Sigma_{nn}}}\right) - \mathcal{N}\left(\frac{\nu_n^{sup} - \bar{\nu}_n}{\sqrt{\Sigma_{nn}}}\right)}{\Phi\left(\frac{\nu_n^{sup} - \bar{\nu}_n}{\sqrt{\Sigma_{nn}}}\right) - \Phi\left(\frac{\nu_n^{inf} - \bar{\nu}_n}{\sqrt{\Sigma_{nn}}}\right)} \sqrt{\Sigma_{nn}} \tag{S19}$$

$$\langle \nu_n^2 \rangle_{Q^{(n)}} - \langle \nu_n \rangle_{Q^{(n)}}^2 = \Sigma_{nn} \left[1 + \frac{\frac{\nu_n^{inf} - \bar{\nu}_n}{\Sigma_{nn}} \mathcal{N}\left(\frac{\nu_n^{inf} - \bar{\nu}_n}{\sqrt{\Sigma_{nn}}}\right) - \frac{\nu_n^{sup} - \bar{\nu}_n}{\sqrt{\Sigma_{nn}}} \mathcal{N}\left(\frac{\nu_n^{sup} - \bar{\nu}_n}{\sqrt{\Sigma_{nn}}}\right)}{\Phi\left(\frac{\nu_n^{sup} - \bar{\nu}_n}{\sqrt{\Sigma_{nn}}}\right) - \Phi\left(\frac{\nu_n^{inf} - \bar{\nu}_n}{\sqrt{\Sigma_{nn}}}\right)} + \right. \tag{S20}$$

$$\left. - \left(\frac{\mathcal{N}\left(\frac{\nu_n^{inf} - \bar{\nu}_n}{\sqrt{\Sigma_{nn}}}\right) - \mathcal{N}\left(\frac{\nu_n^{sup} - \bar{\nu}_n}{\sqrt{\Sigma_{nn}}}\right)}{\Phi\left(\frac{\nu_n^{sup} - \bar{\nu}_n}{\sqrt{\Sigma_{nn}}}\right) - \Phi\left(\frac{\nu_n^{inf} - \bar{\nu}_n}{\sqrt{\Sigma_{nn}}}\right)} \right)^2 \right] \tag{S21}$$

Unfortunately when $\Sigma_{nn} \to 0$ and thus $x \to +\infty$ several numeric issues occur when we compute (S19),(S20). We propose an expansion up to the 5th order of these equations to overcome this problem (see details in Supplementary note 5).

Supplementary note 3. Moments of $Q(\boldsymbol{\nu}|\boldsymbol{b})$

Let us compute $\langle \nu_n \rangle_Q$ and $\langle \nu_n^2 \rangle_Q$ as

$$\langle \nu_n \rangle_Q = \frac{1}{\tilde{Z}_Q} \int d\nu_n \nu_n \phi_n(\nu_n) \int \prod_{m \neq n} d\nu_m e^{-\frac{1}{2}(\boldsymbol{\nu}-\bar{\boldsymbol{\nu}})^T \boldsymbol{\Sigma}^{-1}(\boldsymbol{\nu}-\bar{\boldsymbol{\nu}})} \tag{S22}$$

$$\langle \nu_n^2 \rangle_Q = \frac{1}{\tilde{Z}_Q} \int d\nu_n \nu_n^2 \phi_n(\nu_n) \int \prod_{m \neq n} d\nu_m e^{-\frac{1}{2}(\boldsymbol{\nu}-\bar{\boldsymbol{\nu}})^T \boldsymbol{\Sigma}^{-1}(\boldsymbol{\nu}-\bar{\boldsymbol{\nu}})} \tag{S23}$$

Integrating out the multivariate Gaussian we obtain

$$\langle \nu_n \rangle_Q = \frac{1}{\tilde{Z}_Q} \int d\nu_n \nu_n q_n(\nu_n) \tag{S24}$$

$$\langle \nu_n^2 \rangle_Q = \frac{1}{\tilde{Z}_Q} \int d\nu_n \nu_n^2 q_n(\nu_n) \tag{S25}$$

where $q_n(\nu_n)$ is the marginal probability function

$$q_n(\nu_n) \propto e^{-\frac{(\nu_n - a_n)^2}{2d_n}} e^{-\frac{(\nu_n - \bar{\nu}_n)^2}{2\Sigma_{nn}}} \tag{S26}$$

The proportional sign denotes that the normalization constant is missing. Remembering that the product of two Gaussian distributions satisfy

$$\mathcal{N}(x|\mu_1, \sigma_1)\mathcal{N}(x|\mu_2, \sigma_2) = \mathcal{N}(x|\mu, \sigma)$$

where

$$\begin{cases} \frac{\mu}{\sigma} &= \frac{\mu_1}{\sigma_1} + \frac{\mu_2}{\sigma_2} \\ \frac{1}{\sigma} &= \frac{1}{\sigma_1} + \frac{1}{\sigma_2} \end{cases}$$

In our case, we obtain the following result for the first and second (connected) moment of (S26):

$$\begin{cases} \langle \nu_n^2 \rangle_Q - \langle \nu_n \rangle_Q^2 &= \frac{1}{\frac{1}{d_n} + \frac{1}{\Sigma_{nn}}} \\ \langle \nu_n \rangle_Q &= \left(\frac{1}{d_n} + \frac{1}{\Sigma_{nn}}\right)^{-1} \left(\frac{a_n}{d_n} + \frac{\bar{\nu}_n}{\Sigma_{nn}}\right) \end{cases} \tag{S27}$$

Supplementary note 4. Fast computation of $\boldsymbol{\Sigma}$ and $\bar{\boldsymbol{\nu}}$

Each time we update the parameters of one ϕ_n we need to build a new matrix $\mathbf{D}$ and solve the system of equations in Eq. (16) which requires the inversion of a big matrix of dimension $N \times N$. Globally we need to invert N times a large matrix per iteration which severely affects the computational time. Here we present a scheme by which we can invert one large matrix per iteration.

Let us define $\mathbf{D}'$ a diagonal matrix of elements $D'_{nn} = \frac{1}{d_n}$ and $\boldsymbol{\Sigma}'$, $\bar{\boldsymbol{\nu}}'$ the solutions of

$$\begin{cases} \boldsymbol{\Sigma}'^{-1} &= \beta \mathbf{S}^T \mathbf{S} + \mathbf{D}' \\ \bar{\boldsymbol{\nu}}' &= \boldsymbol{\Sigma}' \left(\beta \mathbf{S}^T \boldsymbol{b} + \mathbf{D}' \boldsymbol{a}\right) \end{cases} \tag{S28}$$

We aim at determining the values of $\mathbf{\Sigma}$ and $\bar{\boldsymbol{\nu}}$ entering in the computation of a_n and d_n as functions of $\mathbf{\Sigma}^{'}$and $\bar{\boldsymbol{\nu}}^{'}$that can be computed per each iteration. Let us write for each flux n the respective $\mathbf{D}$ matrix as $\mathbf{D} =\mathbf{D'} - \frac{1}{d_n}\boldsymbol{e}_n\boldsymbol{e}_n^T$ that must satisfy

$$\begin{cases} \left(\beta\mathbf{S}^T\mathbf{S}+\mathbf{D}\right)\bar{\boldsymbol{\nu}} & = \beta\mathbf{S}^T\boldsymbol{b}+\mathbf{D}\boldsymbol{a} \\ \left(\beta\mathbf{S}^T\mathbf{S}+\mathbf{D}^{'}\right)\bar{\boldsymbol{\nu}}^{'} & = \beta\mathbf{S}^T\boldsymbol{b}+\mathbf{D}^{'}\boldsymbol{a} \end{cases} \tag{S29}$$

Take the first equation in (S29) and subtract to the second:

$$\left(\beta\mathbf{S}^T\mathbf{S}+\mathbf{D}^{'}\right)\left(\bar{\boldsymbol{\nu}}-\bar{\boldsymbol{\nu}}^{'}\right)-\frac{1}{d_n}\boldsymbol{e}_n\boldsymbol{e}_n^T\bar{\boldsymbol{\nu}} = -\frac{1}{d_n}\boldsymbol{e}_n\boldsymbol{e}_n^T\boldsymbol{a} \tag{S30}$$

$$\left(\beta\mathbf{S}^T\mathbf{S}+\mathbf{D}^{'}\right)^{-1}\left(-\frac{1}{d_n}a_n\boldsymbol{e}_n+\frac{1}{d_n}\bar{\nu}_n\boldsymbol{e}_n\right)+\bar{\boldsymbol{\nu}}^{'} = \bar{\boldsymbol{\nu}} \tag{S31}$$

where it is possible to extract the $\bar{\nu}_n$ component as

$$\bar{\nu}_n\left[1-\left(\beta\mathbf{S}^T\mathbf{S}+\mathbf{D}^{'}\right)^{-1}_{nn}\frac{1}{d_n}\right] = -D^{'}_{nn}a_n\left(\beta\mathbf{S}^T\mathbf{S}+\mathbf{D}^{'}\right)^{-1}_{nn}+\bar{\nu}^{'}_n \tag{S32}$$

$$\bar{\nu}_n = \frac{-\frac{1}{d_n}a_n\left(\beta\mathbf{S}^T\mathbf{S}+\mathbf{D}^{'}\right)^{-1}_{nn}+\bar{\nu}^{'}_n}{1-\left(\beta\mathbf{S}^T\mathbf{S}+\mathbf{D}'\right)^{-1}_{nn}\frac{1}{d_n}} \tag{S33}$$

Equivalently the diagonal elements of $\mathbf{\Sigma}$ satisfying $\mathbf{\Sigma}^{-1} = \beta\mathbf{S}^T\mathbf{S}+\mathbf{D}$ can be computed as follows. We define $\boldsymbol{x}$ the solution of equation$\mathbf{\Sigma}^{-1}\boldsymbol{x} = \boldsymbol{e}_n$ such that $\boldsymbol{x}$ is the nth column of $\mathbf{\Sigma}$; thus $x_n = \Sigma_{nn}$. Now consider the homogeneous equation $\mathbf{\Sigma}^{'-1}\boldsymbol{x}^{'} = \mathbf{0}$ for $\mathbf{\Sigma}^{'-1} = \beta\mathbf{S}^t\mathbf{S}+\mathbf{D}^{'}$which surely has solution $\boldsymbol{x}^{'}$=$\mathbf{0}$. We write the system of equations:

$$\begin{cases} \left(\beta\mathbf{S}^T\mathbf{S}+\mathbf{D}\right)\boldsymbol{x} & = \boldsymbol{e}_n \\ \left(\beta\mathbf{S}^T\mathbf{S}+\mathbf{D}^{'}\right)\boldsymbol{x}^{'} & = \boldsymbol{e}_n \end{cases} \tag{S34}$$

and we proceed with the same argument as before. Take the first equation and subtract the second

$$\left(\beta\mathbf{S}^T\mathbf{S}+\mathbf{D}^{'}-\frac{1}{d_n}\boldsymbol{e}_n\boldsymbol{e}_n^T\right)\boldsymbol{x}-\left(\beta\mathbf{S}^T\mathbf{S}+\mathbf{D}^{'}\right)\boldsymbol{x}^{'} = \boldsymbol{e}_n \tag{S35}$$

$$\left(\beta\mathbf{S}^T\mathbf{S}+\mathbf{D}^{'}\right)\left(\boldsymbol{x}-\boldsymbol{x}^{'}\right)-\frac{1}{d_n}x_n\boldsymbol{e}_n = \boldsymbol{e}_n \tag{S36}$$

$$\left(\beta\mathbf{S}^T\mathbf{S}+\mathbf{D}^{'}\right)\boldsymbol{x}^{'}+\boldsymbol{e}_n+\frac{1}{d_n}x_n\boldsymbol{e}_n = \left(\beta\mathbf{S}^T\mathbf{S}+\mathbf{D}^{'}\right)\boldsymbol{x} \tag{S37}$$

$$\boldsymbol{x}^{'}+\left(1+\frac{1}{d_n}x_n\right)\left(\beta\mathbf{S}^T\mathbf{S}+\mathbf{D}^{'}\right)^{-1}\boldsymbol{e}_n = \boldsymbol{x} \tag{S38}$$

Since $\boldsymbol{x}^{'} = \mathbf{0}$, the n^{th}component of $\boldsymbol{x}$ will be:

$$x_n = \left(1+\frac{1}{d_n}x_n\right)\left(\beta\mathbf{S}^T\mathbf{S}+\mathbf{D}^{'}\right)^{-1}_{nn} \tag{S39}$$

$$x_n = \frac{\left(\beta\mathbf{S}^T\mathbf{S}+\mathbf{D}^{'}\right)^{-1}_{nn}}{1-\left(\beta\mathbf{S}^T\mathbf{S}+\mathbf{D}'\right)^{-1}_{nn}\frac{1}{d_n}}. \tag{S40}$$

Finally

$$\begin{cases} \Sigma_{nn} & = \frac{\Sigma^{'}_{nn}}{1-\Sigma^{'}_{nn}\frac{1}{d_n}} \\ \bar{\nu}_n & = \frac{-\frac{1}{d_n}a_n\Sigma^{'}_{nn}+\bar{\nu}^{'}_n}{1-\Sigma^{'}_{nn}\frac{1}{d_n}} \end{cases} \tag{S41}$$

Components of $\bar{\boldsymbol{\nu}}$ different from $\bar{\nu}_n$ and all non-diagonal entries of $\mathbf{\Sigma}$ can be computed following the same strategy; we do not report their expression here since the update rules of a_n and d_n in Eq. (18) only depends on terms in (S41).

Supplementary note 5. Asymptotic expansion of the first two moments of the tilted distribution

As already remarked in Supplementary note 2, the computation of the first two moments of the tilted distribution $Q^{(n)}$ defined in Eq. (15) turns out to be numerically difficult to compute in particular in cases when we need to evaluate integrals over the tails of the distributions. To overcome such difficulties, we resorted to an asymptotic expansion to the 5th order which accounts for both accuracy and numerical stability in all conditions analyzed in our tests. The idea is to start by noting that up to the required precision, in the limit $x \to \infty$, $\Phi(x) \simeq \frac{1}{2} - \mathcal{N}(x)\left(\frac{1}{x} - \frac{1}{x^3} + \frac{3}{x^5} - o\left(\frac{1}{x^7}\right)\right)$ so that:

$$\frac{\phi(x_0)-\phi(x_1)}{\Phi(x_1)-\Phi(x_0)} = \frac{e^{-\frac{x_0^2}{2}} - e^{-\frac{x_1^2}{2}}}{e^{-\frac{x_0^2}{2}}\left(\frac{1}{x_0} - \frac{1}{x_0^3} + \frac{3}{x_0^5}\right) - e^{-\frac{x_1^2}{2}}\left(\frac{1}{x_1} - \frac{1}{x_1^3} + \frac{3}{x_0^5}\right)}$$

$$= \frac{x_0^5 x_1^5\left(1 - e^{\frac{x_1^2-x_0^2}{2}}\right)}{e^{\frac{x_1^2-x_0^2}{2}} x_1^5(-3+x_0^2+x_0^4) + x_0^5(3-x_1^2+x_1^4)} \tag{S42}$$

$$= \begin{cases} \frac{x_0^5}{3-x_0^2-x_0^4} & \text{for } (x_1^2-x_0^2) \to \infty \\ \frac{x_1^5}{3-x_1^2+x_1^4} & \text{for } (x_1^2-x_0^2) \to -\infty \end{cases} \tag{S43}$$

$$\frac{x_0\phi(x_0)-x_1\phi(x_1)}{\Phi(x_1)-\Phi(x_0)} = \frac{x_0 e^{-\frac{x_0^2}{2}} - x_1 e^{-\frac{x_1^2}{2}}}{e^{-\frac{x_0^2}{2}}\left(\frac{1}{x_0} - \frac{1}{x_0^3} + \frac{3}{x_0^5}\right) - e^{-\frac{x_1^2}{2}}\left(\frac{1}{x_1} - \frac{1}{x_1^3} + \frac{3}{x_1^5}\right)}$$

$$= \frac{x_0^5 x_1^5\left(x_1 - x_0 e^{\frac{x_1^2-x_0^2}{2}}\right)}{e^{\frac{x_1^2-x_0^2}{2}} x_1^5(-3+x_0^2-x_0^4) + x_0^5(3-x_1^2+x_1^4)} \tag{S44}$$

$$= \begin{cases} \frac{x_0^6}{3-x_0^2-x_0^4} & \text{for } (x_1^2-x_0^2) \to \infty \\ \frac{x_1^6}{3-x_1^2-x_1^4} & \text{for } (x_1^2-x_0^2) \to -\infty \end{cases} \tag{S45}$$

Taking in consideration the expressions above and defining $x_0 = \frac{\nu_n^{inf} - \bar{\nu}_n}{\sqrt{\Sigma_{nn}}}$, $x_1 = \frac{\nu_n^{sup} - \bar{\nu}_n}{\sqrt{\Sigma_{nn}}}$, $s = \mathrm{sign}(\mathrm{x_0 x_1})$, $m = \min(|x_0|, |x_1|)$, $\Delta^2 = \frac{x_1^2 - x_0^2}{2}$, and calling

$$\gamma = \gamma(x_0, x_1) = \frac{x_0^5 x_1^5}{e^{\frac{x_1^2-x_0^2}{2}} x_1^5(-3+x_0^2+x_0^4) + x_0^5(3-x_1^2+x_1^4)}$$

we can finally approximate the two first moments of the tilted distribution in the following manner:

$$\langle\nu\rangle_{Q^{(n)}} = \bar{\nu} + \sqrt{\Sigma_{nn}} \cdot \begin{cases} \frac{\mathcal{N}(x_0)-\mathcal{N}(x_1)}{\Phi(x_1)-\Phi(x_0)} & \text{for m} \le 6 \text{ or s} = -1 \\ \gamma\left(1 - e^{\frac{x_1^2-x_0^2}{2}}\right) & \text{for m} \ge 6, \text{ s} = 1, \Delta^2 < 40 \\ \frac{x_0^5}{3-x_0^2-x_0^4} & \text{for m} \ge 6, \text{ s} = 1, \Delta^2 \ge 40 \end{cases}$$

$$\langle\nu^2\rangle_{Q^{(n)}} - \langle\nu\rangle_{Q^{(n)}}^2 = \Sigma_{nn} \cdot \begin{cases} 1 + \frac{x_0\mathcal{N}(x_0)-x_1\mathcal{N}(x_1)}{\Phi(x_1)-\Phi(x_0)} - \left(\frac{\mathcal{N}(x_0)-\mathcal{N}(x_1)}{\Phi(x_1)-\Phi(x_0)}\right)^2 & \text{for } m \le 6 \text{ } or \text{ } s = -1 \\ 1 + \gamma\left(x_1 - x_0 e^{\frac{x_1^2-x_0^2}{2}}\right) - \gamma^2\left(1 - e^{\frac{x_1^2-x_0^2}{2}}\right)^2 & \text{for } m \ge 6, \ s = 1, \ \Delta^2 < 40 \\ 1 + \frac{x_0^6}{3-x_0^2-x_0^4} - \left(\frac{x_0^5}{3-x_0^2-x_0^4}\right)^2 & \text{for } m \ge 6, \ s = 1, \ \Delta^2 \ge 40 \end{cases}$$

Note that the generating series for Φ is of alternate signs and one can easily see that upon considering only the 3rd order, the variance might turn to be negative. To overcome this difficulty, only terms of order $1, 5, \ldots, 4n+1$ must be considered, and so the next useful approximation is of order 9th.

Supplementary note 6. Weighted Hit-and-Run

Hit-and-Run is a Monte Carlo method that aims at uniformly sample the feasible configuration space of fluxes. To add a non-uniform prior such as $g(\nu_i; a_i, d_i)$ in Eq. (19) , one should resort to an importance sampling generalization of HR (see e.g. [1]). However, the determination of the parameters a_i and d_i must be done by multiple HR convergences in some sort of gradient descent scheme (in a procedure similar to Boltzmann learning), which is deemed to be too time consuming.

A seemingly simpler alternative is to perform a re-weighting of the configurations explored by the uniform sampling in a way that the HR marginal of the flux fits the experimental data. Calling ν_i the experimentally known flux and defining the re-weighting function as $g(\nu_i; a_i, d_i)$, our scope is to tune a and b to reproduce the experimental marginal. More formally $g(\nu_i; a_i, d_i)$ is the exponential function of the unknown Lagrange multipliers enforcing the constraint on the fixed marginal as the one introduced in Eq. (19). One way of determining the two parameters and of performing the sampling can be the following. The empirical first and second moments of the re-weighted distribution will read

$$\begin{aligned} \langle \nu_i \rangle_g &\simeq \sum_{\alpha=1}^{A} \nu_{i,\alpha} \frac{g(\nu_{i,\alpha}; a_i, d_i)}{W} \\ \langle \nu_i^2 \rangle_g &\simeq \sum_{\alpha=1}^{A} \nu_{i,\alpha}^2 \frac{g(\nu_{i,\alpha}; a_i, d_i)}{W} \end{aligned} \tag{S46}$$

where the index α runs over all sampled configurations and $W = \sum_\alpha g(\nu_{i,\alpha}; a_i, d_i)$ is the normalization constant. This is a 2×2 system that needs to be solved for variables a_i, d_i.

However, we will show in the following that the approximation in Eq. S46 is normally too rough to expect good results with a reasonable number of sample points in most cases. To give an example of the reliability of this procedure let us fix two marginals of our choice for the biomass of a population of Escherichia Coli described by the modified *iJR904* model introduced in section “Results” of the main text. The distribution of the unconstrained biomass flux in this network is roughly a Gaussian distribution with standard deviation $2 \cdot 10^{-2}$ and mean $\mu = 0.03$ as shown in Supplementary figure 1 (a). We will attempt to constraint the system to two different “observed” biomass fluxes, both with standard deviation 10^{-2} and means 0.09 and 0.2 respectively.

We start by computing a uniformly weighted sampling set with standard HR. We performed this with three different sets sizes $T = \{2.56 \cdot 10^7,\ 1.02 \cdot 10^8,\ 4.10 \cdot 10^8\}$. In each of the two cases, we also apply constrained EP to fix the marginal distribution to the observed one, obtaining parameters $a_i^{EP(0.09)}, d_i^{EP(0.09)}$ and $a_i^{EP(0.2)}, d_i^{EP(0.2)}$. Now we can perform the re-weighting of the configurations according to the functions $g\left(\nu_i; a_i^{EP(0.09)}, d_i^{EP(0.09)}\right)$ and $g\left(\nu_i; a_i^{EP(0.2)}, d_i^{EP(0.2)}\right)$. We show in Supplementary figure 1 the re-weighted marginals of the biomass flux (blue, green and yellow bars) along with the Gaussian distributions with mean 0.09 (Supplementary figure 1 (b)) and the one with mean 0.2 (Supplementary figure 1 (c)) that we would like to retrieve (red line). In Supplementary figure 1 (b) we can notice that as we increase the number of sampled points, the re-weighted marginal very slowly approaches the desired one; differently in Supplementary figure 1 (c) HR estimate fails to retrieve the fixed profile, indicating that a much larger set of uniform sampling points would be needed.

The reason is that in the second case, being the mean in the unconstrained case equal to 0.03, for an exponentially overwhelming fraction of the HR points the value of the flux ν_i is far from the mean value of the experimental distribution (and thus the associated weight $g(\nu_i; a, b)$ is exponentially small). As a consequence the number of sampling points needed to reasonably sample the constrained distribution becomes extremely large. This is what happens to the experimental growth rate described in the “Results” section that cannot be recovered by this method in a feasible time.

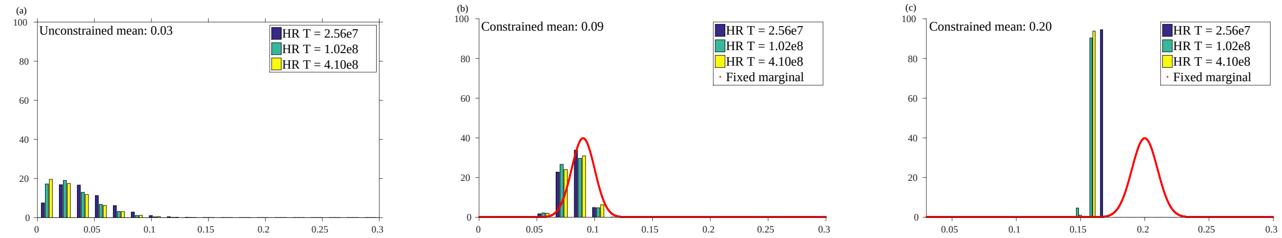

Supplementary figure 1: Marginal probability densities for the biomass flux computed through the re-weighting procedure (blue bars) and the fixed ones (red line) in two cases: (b) the fixed profile has mean 0.09 while in (c) the mean has been shifted to 0.2. Fig. (a) shows the marginal in the unconstrained case.

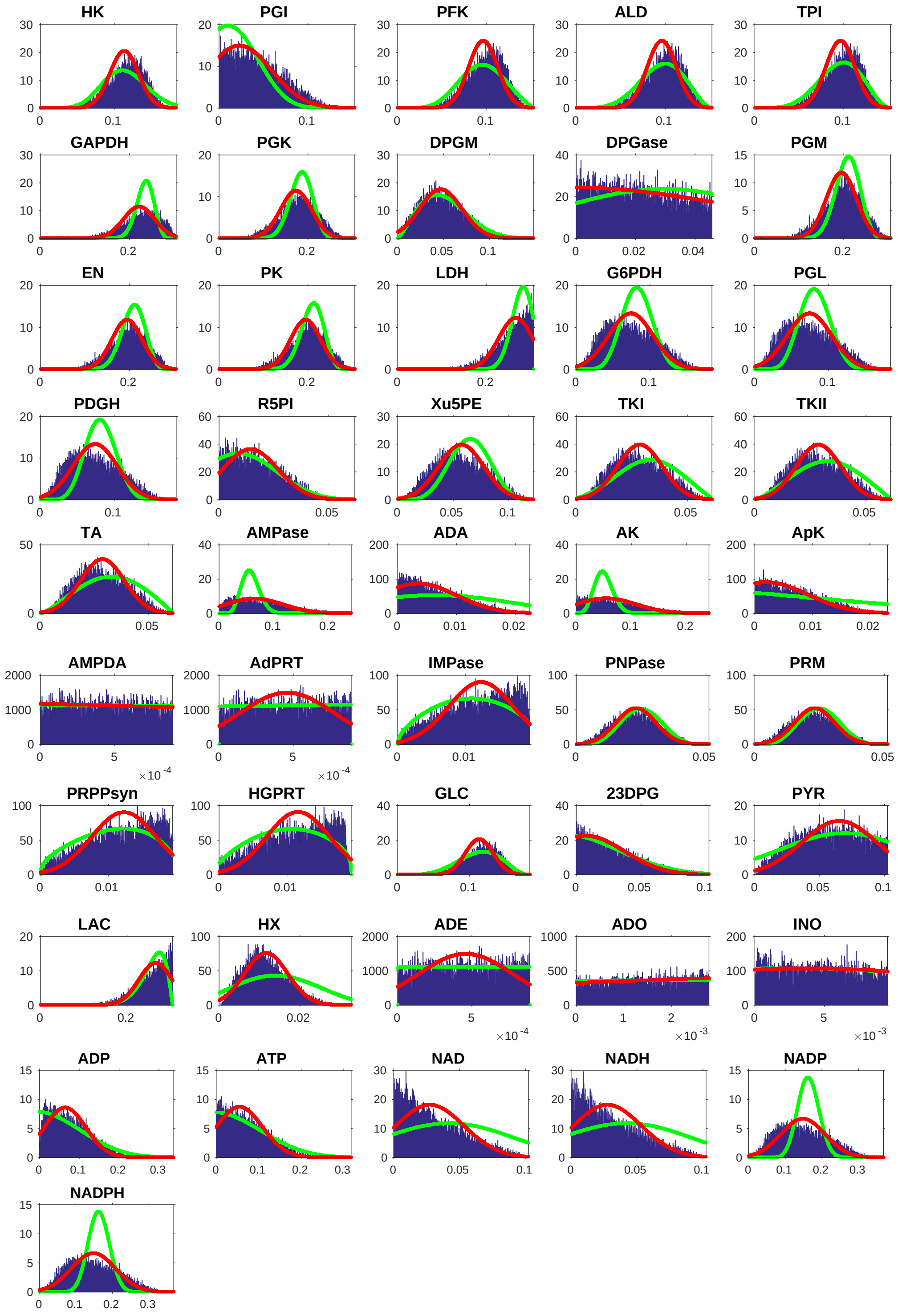

Supplementary figure 2: Marginals of the entire set of fluxes of red blood cell. The blue bars are obtained through HR sampling for $T \sim 10^8$ explored configurations; the green line is the prediction of the Belief propagation (BP) algorithm in [2] while the red line denotes the results of our EP algorithm.

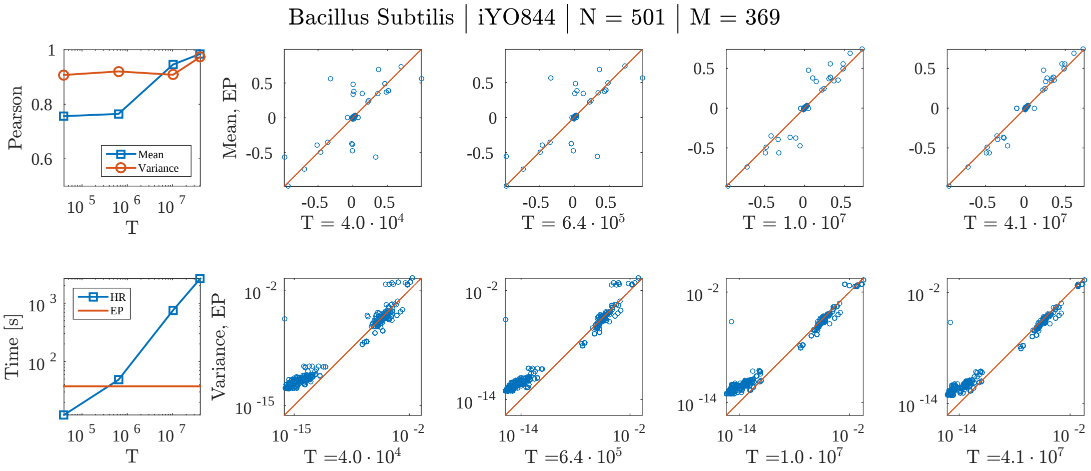
Bacillus Subtilis | iYO844 | N = 501 | M = 369
Pearson
Mean
Variance
T
Time [s]
HR
EP
Mean, EP
Variance, EP
T = 4.0 · 10^4
T = 6.4 · 10^5
T = 1.0 · 10^7
T = 4.1 · 10^7

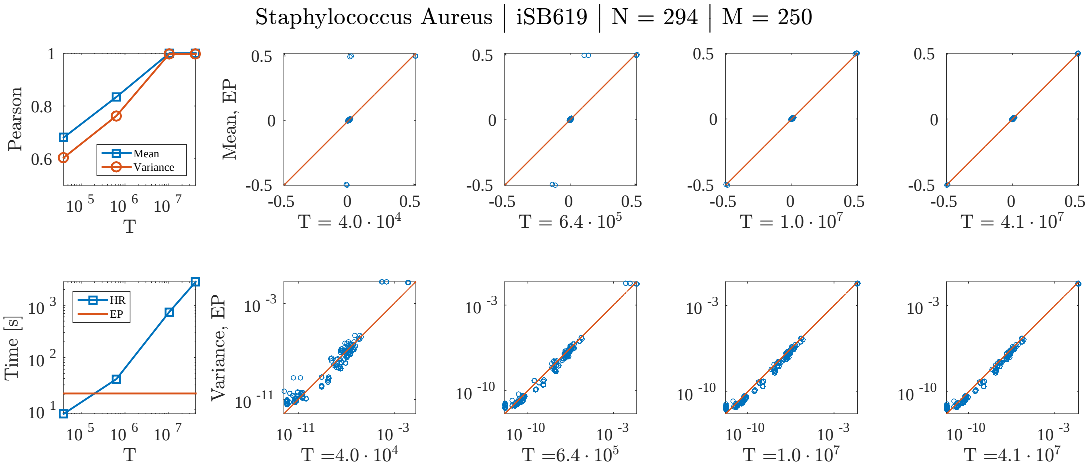
Staphylococcus Aureus | iSB619 | N = 294 | M = 250
Pearson
Mean
Variance
T
Time [s]
HR
EP
Mean, EP
Variance, EP
T = 4.0 · 10^4
T = 6.4 · 10^5
T = 1.0 · 10^7
T = 4.1 · 10^7

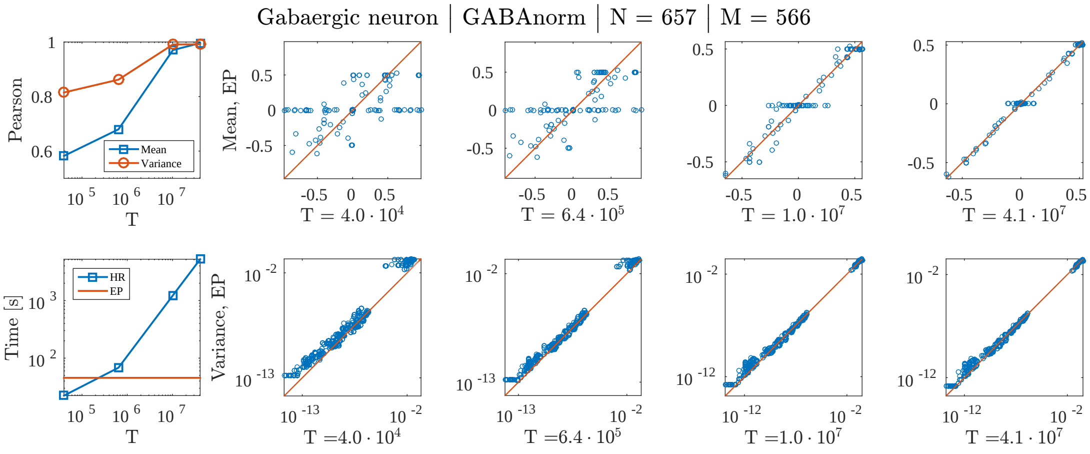
Gabaergic neuron | GABAnorm | N = 657 | M = 566
Pearson
Mean
Variance
T
Time [s]
HR
EP
Mean, EP
Variance, EP
T = 4.0 · 10^4
T = 6.4 · 10^5
T = 1.0 · 10^7
T = 4.1 · 10^7

Glutamatergic neuron | GLUnorm | N = 657 | M = 567

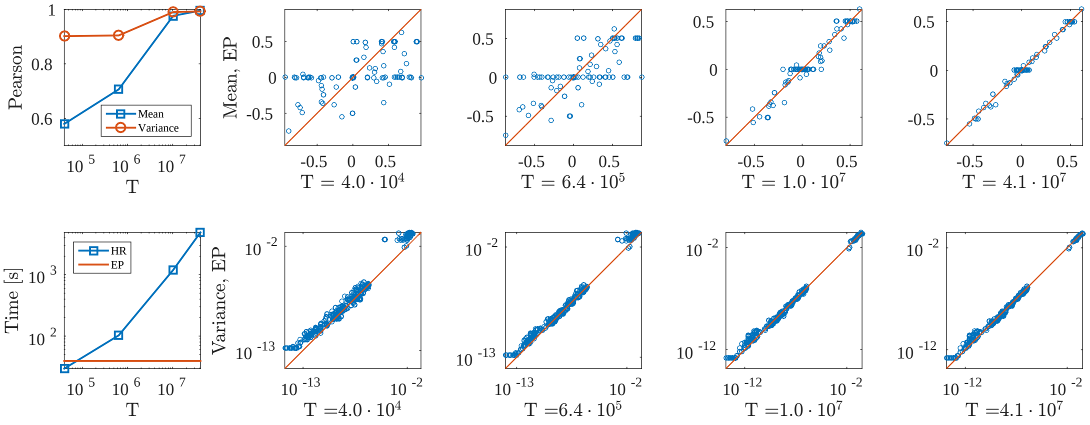

Saccharomyces Cerevisiae | iND750 | N = 389 | M = 296

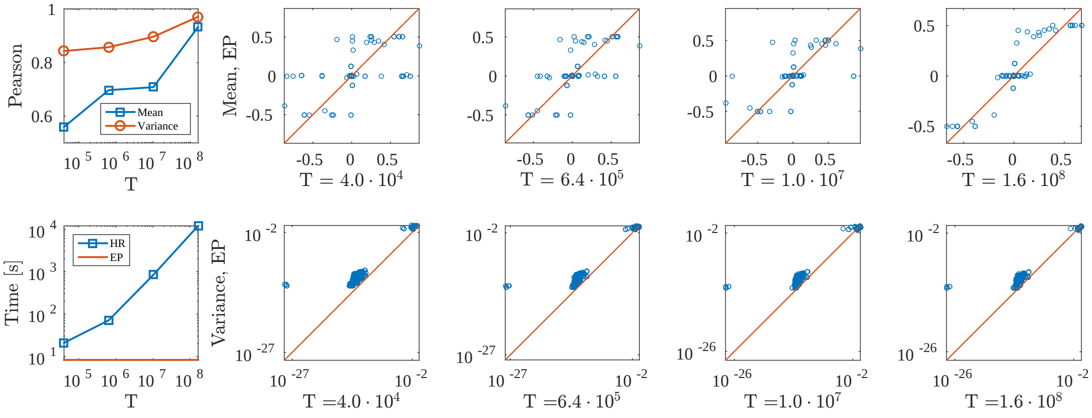

Mycobacterium Tuberculosis | iNJ661 | N = 414 | M = 316

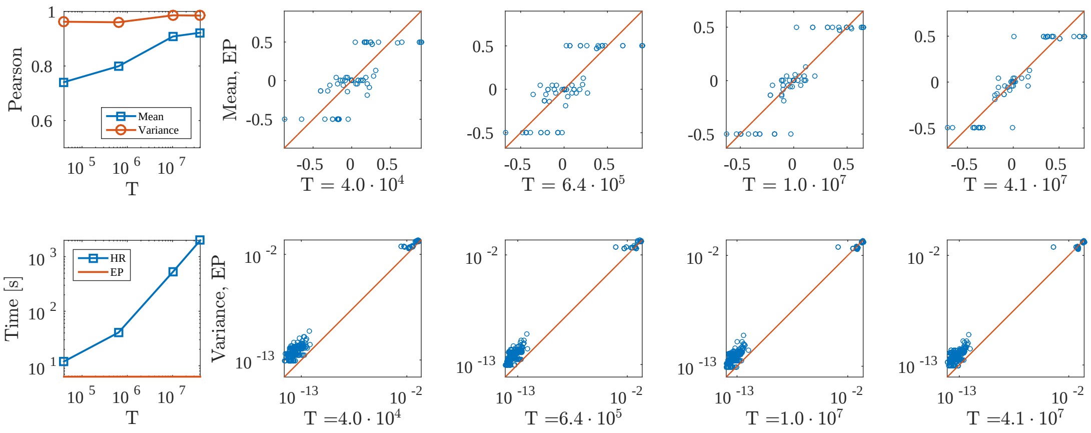

Methanosarcina Barkeri | iAF692 | N = 131 | M = 117

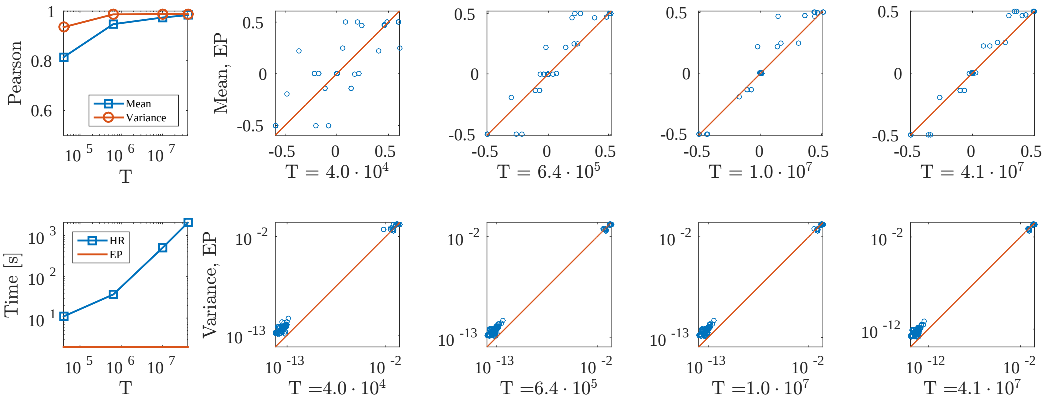

Helicobacter Pylori | iIT341 | N = 210 | M = 175

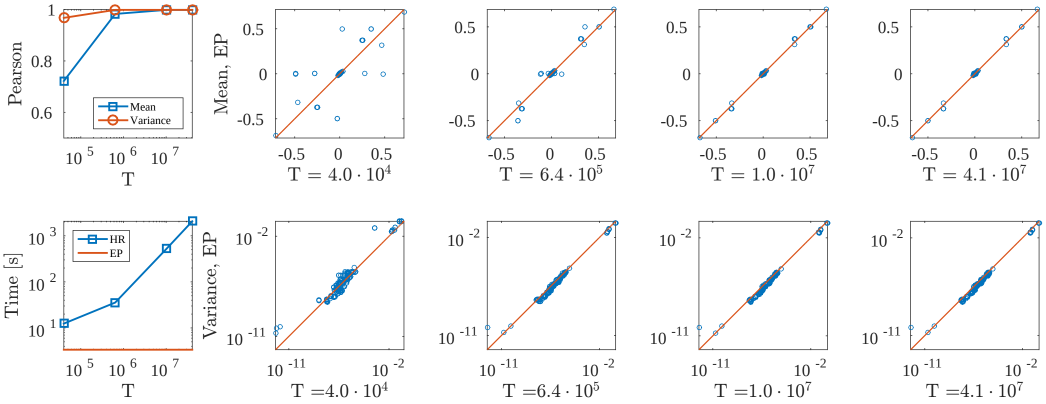

Clostridium Thermocellum | iSR432 | N = 399 | M = 323

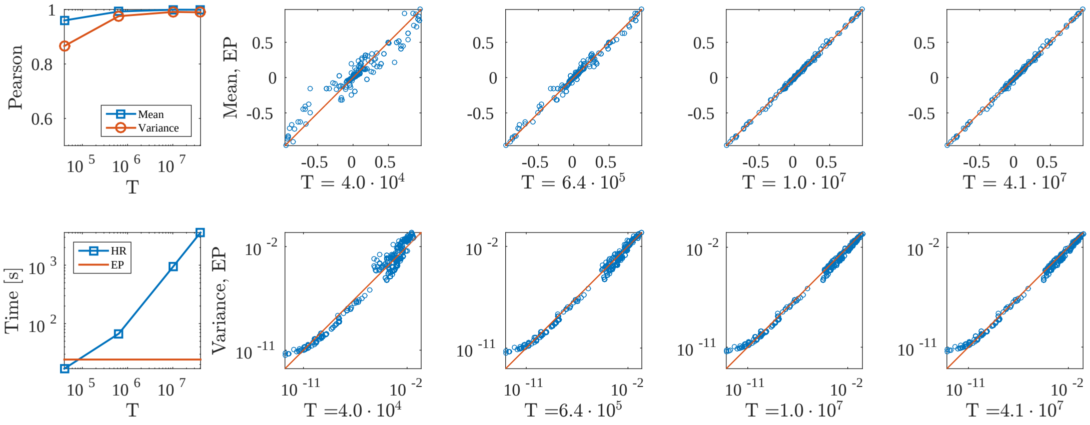

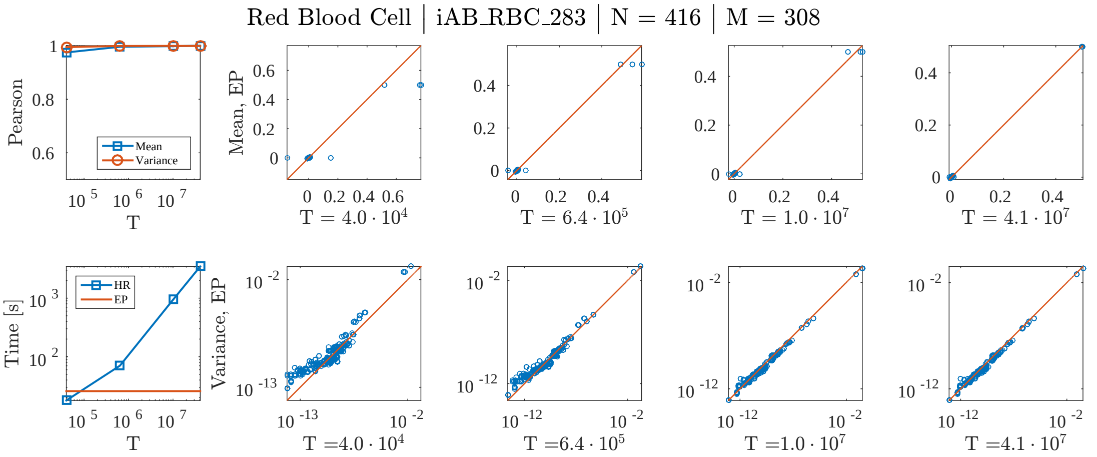

Supplementary figure 3: Comparison between HR and EP for several models of large scale metabolic networks available in the Bigg Models database [3].

SUPPLEMENTARY REFERENCES

[1] Ming-Hui Chen and Bruce W. Schmeiser. General Hit-and-Run Monte Carlo sampling for evaluating multidimensional integrals. *Operations Research Letters*, 19(4):161–169, October 1996.

[2] J. Fernandez-de Cossio-Diaz and R. Mulet. Fast inference of ill-posed problems within a convex space. *Journal of Statistical Mechanics: Theory and Experiment*, 2016(7):073207, 2016.

[3] Zachary A King, Justin Lu, Andreas Dräger, Philip Miller, Stephen Federowicz, Joshua A Lerman, Ali Ebrahim, Bernhard O Palsson, and Nathan E Lewis. Bigg models: A platform for integrating, standardizing and sharing genome-scale models. *Nucleic acids research*, 44(D1):D515–D522, 2016.
